# Supplementary material for: Landscape-scale analysis of raccoon rabies surveillance reveals different drivers of disease dynamics across latitude
Source: PLoS Negl Trop Dis. 2025 Sep 26;19(9):e0013581. doi: 10.1371/journal.pntd.0013581 (PMC12500145; doi:10.1371/journal.pntd.0013581)
Supplement: S1 Text — (PDF) [file pntd.0013581.s001.pdf]

S1 Text: Landscape-scale analysis of raccoon rabies surveillance reveals different drivers of disease dynamics across latitude

Meggan E Craft Matthew Michalska-Smith Amy J Davis
Amy T Gilbert Richard Chipman Jordona Kirby
Kathleen M Nelson Xiaoyue Ma Ryan Wallace Grace Miller
Kim M Pepin

In the main text of this work, we analyze the spatio-temporal distribution of raccoon rabies across the eastern United States. In particular, we focus on the role of latitude and urbanness (using human population density as a proxy) on the likelihood of rabies being detected in a given county and month. We additionally consider the role of disease detection within spatial buffers and temporal lags, as well as the role of habitat coverage and the number of samples submitted for testing. We find significant interactions between latitude and month, suggesting a seasonality effect at northern latitudes, and between latitude and urbanness, hinting at possible differences in either surveillance or disease dynamics at northern vs. southern latitudes. In this supplement, we present results for two other ways to quantify disease outcomes: proportion positive (the proportion submitted samples found to be positive) and disease persistence (which we quantify as a county having at least 5 months with positive samples over an 11 month lag). We first provide a series of supplementary tables and figures to complement the main text data presentation and results. Next, we detail how we used model selection to develop the aforementioned definition for rabies persistence. Finally we provide analogous results to the main text for each of these additional outcome measures, including a discussion of some limitations associated with using passive surveillance submissions to estimate disease prevalence.

Tables and figures to complement main text

Table A: General linear mixed model results for rabies presence, *i.e.*, the likelihood of having at least one positive rabies sample tested in a given county-month. Spline structure is indicated by the term, with a corresponding number of rows for estimates, standard errors, and p-values. P-value significance is indicated with *, **, and *** indicating p-values less than 0.05, 0.01. and 0.001, respectively.

	Presence			
Term	Estimate	Standard Error	p-value	
Intercept	18.7379	7.7942	0.0162	*
bs(Month, df = 7, degree = 1)	0.0964	0.8173	0.9062	
	-0.3985	0.7914	0.6146	
	-0.4273	0.8863	0.6297	
	0.0846	0.8460	0.9203	
	1.1208	0.8049	0.1638	
	0.3847	0.8310	0.6435	
	0.9037	0.8833	0.3063	
bs(Latitude, df = 7, degree = 1)	-29.7430	8.9233	0.0009	***
	-27.2580	7.7161	0.0004	***
	-18.7958	8.3377	0.0242	*
	-26.2093	8.0042	0.0011	**
	-22.1530	7.8370	0.0047	**
	-22.9659	7.8605	0.0035	**
	-22.6392	7.9777	0.0045	**
bs(Population Density, df = 3, degree = 3)	-71.9401	20.5763	0.0005	***
	25.9715	10.9917	0.0181	*
	-70.2591	25.3656	0.0056	**
Raccoon Favorable Habitat	0.0797	0.1547	0.6067	
Temporal Lag	0.1606	0.0136	0.0000	***
Spatial Effect	0.1999	0.0176	0.0000	***
log(Total Number of Samples Submitted + 1)	5.0347	0.0425	0.0000	***
bs(Latitude, df = 7, degree = 1):bs(Population Density, df = 3, degree = 3)	91.0126	23.9489	0.0001	***
	82.7443	20.4894	0.0001	***
	60.3050	21.8195	0.0057	**
	77.7892	21.0879	0.0002	***
	70.8434	20.6520	0.0006	***
	73.2467	20.6831	0.0004	***
	70.4585	21.1084	0.0008	***
	-36.8348	14.2357	0.0097	**
	-32.9074	11.2707	0.0035	**
	-18.3470	11.5625	0.1126	
	-23.0686	11.3075	0.0413	*
	-24.4629	11.0433	0.0267	*
	-25.4879	11.0313	0.0209	*
	-19.9374	11.4429	0.0814	
	84.5456	32.5201	0.0093	**
	89.7335	25.6956	0.0005	***
	50.2571	26.9300	0.0620	
	68.1324	26.0006	0.0088	**
	66.2969	25.4020	0.0091	**
	70.8256	25.4080	0.0053	**
	63.2166	25.8621	0.0145	*

(Continued on Next Page...)

(continued)

Term	Presence			
	Estimate	Standard Error	p-value	
	0.0503	0.9740	0.9588	
	0.3775	0.9433	0.6890	
	0.2409	1.0459	0.8178	
	-0.1961	1.0014	0.8448	
	-1.2808	0.9572	0.1809	
	-0.3114	0.9871	0.7524	
	-1.0438	1.0587	0.3242	
	-0.3976	0.8469	0.6387	
	0.6127	0.8166	0.4531	
	-0.2571	0.9112	0.7778	
	0.1184	0.8705	0.8919	
	-0.8399	0.8312	0.3123	
	-0.9001	0.8629	0.2969	
	-1.0551	0.9115	0.2470	
	-0.0908	0.8550	0.9154	
	0.1762	0.8279	0.8314	
	0.4487	0.9208	0.6261	
	-0.3010	0.8816	0.7328	
	-0.7628	0.8406	0.3642	
	-0.6452	0.8707	0.4587	
	-0.9066	0.9257	0.3274	
	0.1888	0.8486	0.8240	
	0.8296	0.8203	0.3119	
	-0.4904	0.9156	0.5922	
bs(Month, df = 7, degree = 1):bs(Latitude, df = 7, degree = 1)	-0.0117	0.8752	0.9893	
	-0.7945	0.8341	0.3408	
	-0.4635	0.8632	0.5913	
	-0.9831	0.9191	0.2848	
	-0.1477	0.8448	0.8612	
	0.3789	0.8172	0.6429	
	-0.0731	0.9115	0.9361	
	-0.7500	0.8727	0.3902	
	-1.1835	0.8313	0.1546	
	-0.4230	0.8588	0.6224	
	-0.7713	0.9114	0.3974	
	-0.2471	0.8370	0.7678	
	-0.0896	0.8106	0.9120	
	-2.1967	0.9058	0.0153	*
	-1.0264	0.8654	0.2356	
	-1.2344	0.8237	0.1339	
	-1.0856	0.8516	0.2024	
	-1.3911	0.9051	0.1243	
	-0.0804	0.9436	0.9321	
	-0.0591	0.9164	0.9486	
	-1.0263	1.0086	0.3089	
	-1.1771	0.9743	0.2270	
	-1.3012	0.9263	0.1601	
	-0.9103	0.9667	0.3464	
	-0.5742	1.0140	0.5712	

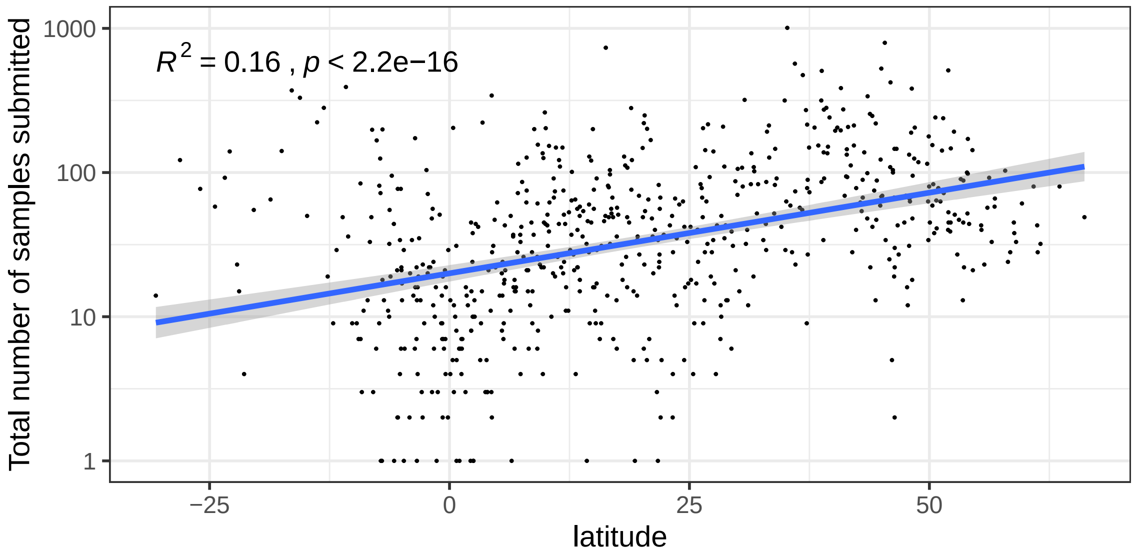

Figure A: There is a significant, positive correlation between the total number of samples submitted in a given county over the timeframe of our dataset and the centroid latitude of the county. Note the vertical axis is log-transformed. R^2 and p-value reported are the result of a Pearson correlation. The blue line is a best-fitting linear model.

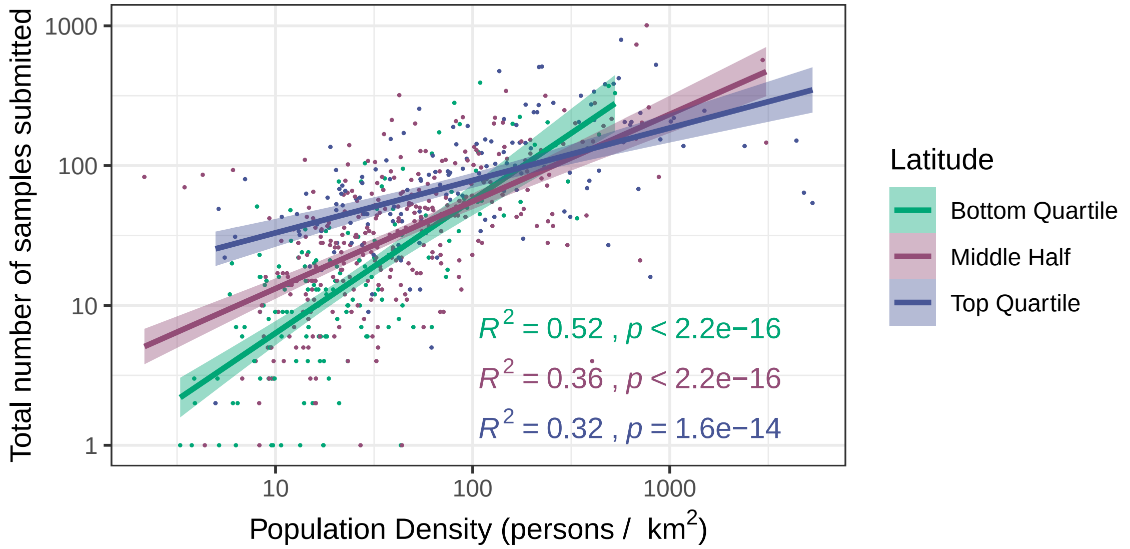

Figure B: Correlation of county human population density and total number of samples submitted in a given county over the timeframe of our dataset. Note both axes are log-scaled. Reported R^2 and p-value correspond to a Pearson correlation of the log-transformed variables.

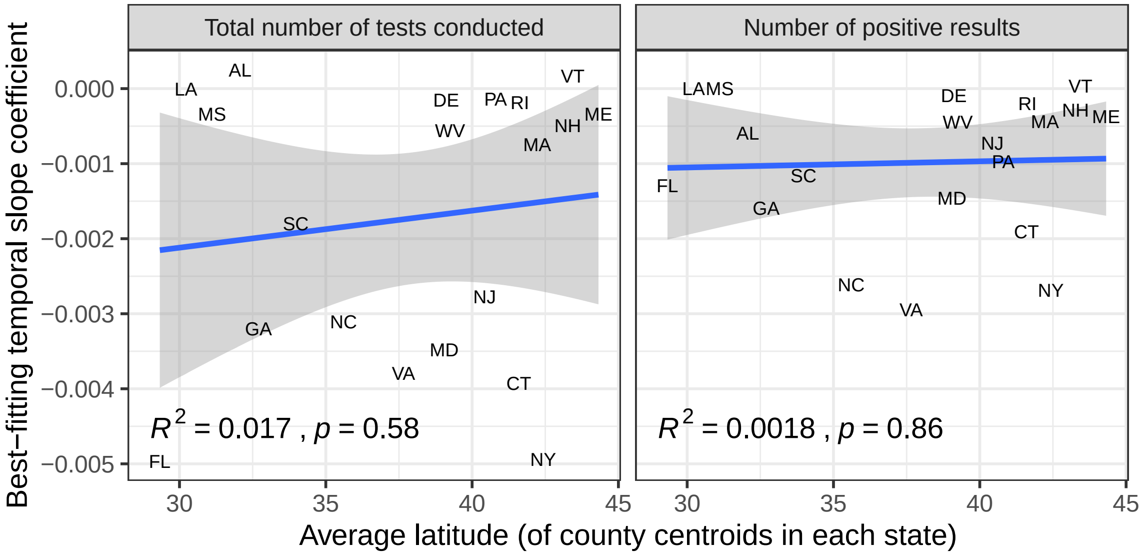

Figure C: Best-fitting linear model slope coefficients for total tests conducted (left) and number of positive results (right) over the thirteen years of our dataset. Linear models were fit to each state's monthly records from 2006-2018, using time as the independent variable. Though many states had negative slopes in their best-fit models, we see no consistent relationship between these slopes and the mean latitude of each state for either tests conducted or positive test results in our dataset.

Model selection for determining definition of persistence

Counties with a "persistent" occurrence of raccoon RABV were defined as at least n months with a positive sample over the last m months, with n and m chosen to meet the following criteria: 1) n greater than 1 and m greater than 3 (to avoid conflicts with our parameters of spatial and temporal lag), 2) between 25-75% of county-months meeting the criteria (to ensure that we would have enough variation in the model), and 3) having the best fit to the data according to AIC, given these constraints. The values that met these criteria were: $n = 5$, $m = 11$.

Analogous tables and figures to main text

Table B: Summary of significant terms for each of the three response measures used in the main text (Presence: the likelihood of having at least one positive test result in a given county-month) and supplementary information (Proportion Positive: the proportion of tests expected to be found positive in a given county-month; Persistence: the likelihood of a county having at least one positive test in each of 5 of the past 11 months). Spline structure is indicated by the term, with a corresponding number of rows for estimates, standard errors, and p-values. n cases where a spline is fit in the model (*i.e.* Month, Latitude, Density, and, consequently, their interaction terms (indicated with ":")), multiple estimates and p-values are associated with each term. Here, we only report the significance of the smallest associated p-value as an indication of the overall significance of that term, with *, **, and *** indicating p-values less than 0.05, 0.01. and 0.001, respectively. The details of each fitted spline can be found in Table A, Table C, and Table D.

Term	Presence	Proportion Positive	Persistence
(Intercept)	*	*	***
Month			
Latitude	***	***	
Population Density	***		
Raccoon Favorable Habitat			
Temporal Lag	***	***	***
Spatial Effect	***	***	***
log(Total Number of Samples Submitted + 1)	***	***	***
Latitude:Population Density	***	*	
Month:Latitude	*	***	*

Table C: General linear mixed model results for rabies proportion positive, *i.e.*, the expected number of positive samples divided by the total number of samples submitted. Spline structure is indicated by the term, with a corresponding number of rows for estimates, standard errors, and p-values. P-value significance is indicated with *, **, and *** indicating p-values less than 0.05, 0.01. and 0.001, respectively.

	Proportion Positive			
Term	Estimate	Standard Error	p-value	
Intercept	-1.0474	0.4174	0.0121	*
bs(Month, df = 6, degree = 1)	0.0096	0.3203	0.9761	
	-0.1038	0.3050	0.7335	
	0.0897	0.3261	0.7833	
	-0.0688	0.3238	0.8317	
	-0.0186	0.2969	0.9500	
	0.0185	0.3044	0.9516	
bs(Latitude, df = 5, degree = 1)	1.4441	0.5115	0.0048	**
	1.1712	0.4273	0.0061	**
	1.5532	0.4458	0.0005	***
	1.6642	0.4363	0.0001	***
	1.7164	0.4937	0.0005	***
Population Density	0.6322	0.7354	0.3899	
Raccoon Favorable Habitat	0.0023	0.0601	0.9691	
Temporal Lag	0.1026	0.0093	0.0000	***
Spatial Effect	0.1332	0.0120	0.0000	***
log(Total Number of Samples Submitted + 1)	-0.1167	0.0266	0.0000	***
bs(Latitude, df = 5, degree = 1):Population Density	-1.9278	0.9160	0.0353	*
	-0.9075	0.7462	0.2239	
	-1.8264	0.7803	0.0193	*
	-1.1518	0.7528	0.1260	
	-0.5217	0.8422	0.5356	

(Continued on Next Page...)

(continued)

Term	Proportion Positive			
	Estimate	Standard Error	p-value	
bs(Month, df = 6, degree = 1):bs(Latitude, df = 5, degree = 1)	0.0477	0.4372	0.9132	
	0.1065	0.4072	0.7936	
	-0.3415	0.4328	0.4301	
	0.3360	0.4341	0.4390	
	-0.0735	0.3986	0.8537	
	-0.0326	0.4101	0.9367	
	-0.0577	0.3336	0.8628	
	0.0749	0.3150	0.8122	
	-0.2170	0.3373	0.5201	
	0.3179	0.3353	0.3431	
	-0.0236	0.3099	0.9394	
	-0.0887	0.3176	0.7800	
	0.1613	0.3564	0.6510	
	0.1919	0.3353	0.5671	
	-0.4244	0.3576	0.2352	
	0.2377	0.3558	0.5042	
	-0.0114	0.3307	0.9726	
	0.0511	0.3416	0.8810	
	-0.1638	0.3379	0.6279	
	-0.2426	0.3194	0.4476	
	-0.7504	0.3412	0.0278	*
	0.0481	0.3392	0.8873	
	-0.1996	0.3133	0.5241	
	-0.1522	0.3220	0.6365	
	-0.2456	0.4266	0.5648	
	-0.7778	0.3950	0.0489	*
	-1.4517	0.4164	0.0005	***
	-0.1622	0.4176	0.6978	
	-0.7280	0.3997	0.0686	
	-0.1398	0.4143	0.7357	

Table D: General linear mixed model results for likelihood of rabies persistence, *i.e.*, the likelihood of a county month meeting the criteria of at least 5 months out of the past 11 having at least one positive test result each. Spline structure is indicated by the term, with a corresponding number of rows for estimates, standard errors, and p-values. P-value significance is indicated with *, **, and *** indicating p-values less than 0.05, 0.01. and 0.001, respectively.

	Persistence			
Term	Estimate	Standard Error	p-value	
Intercept	-16.6653	2.4484	0.0000	***
bs(Month(Monthyear), df = 3, degree = 3)	0.9658	2.0615	0.6394	
	0.1563	1.4917	0.9166	
	0.5296	1.0192	0.6033	
bs(Latitude, df = 2, degree = 2)	5.6320	4.1914	0.1790	
	2.9265	2.0703	0.1575	
Population Density	-2.6297	4.4327	0.5530	
Raccoon Favorable Habitat	-0.2950	0.4087	0.4705	
Spatial Effect	0.2096	0.0433	0.0000	***
Temporal Lag	1.0260	0.0338	0.0000	***
log(Total Number of Samples Submitted Over Previous 11 Months + 1)	4.4344	0.0931	0.0000	***
bs(Latitude, df = 2, degree = 2):Population Density	3.3096	7.7213	0.6682	
	3.5337	3.6974	0.3392	
bs(Month(Monthyear), df = 3, degree = 3):bs(Latitude, df = 2, degree = 2)	-0.7153	3.7956	0.8505	
	-1.4459	2.7560	0.5998	
	-0.6461	1.9170	0.7361	
	-2.2772	1.6433	0.1658	
	2.4093	1.1856	0.0421	*
	-0.5968	0.8109	0.4617	

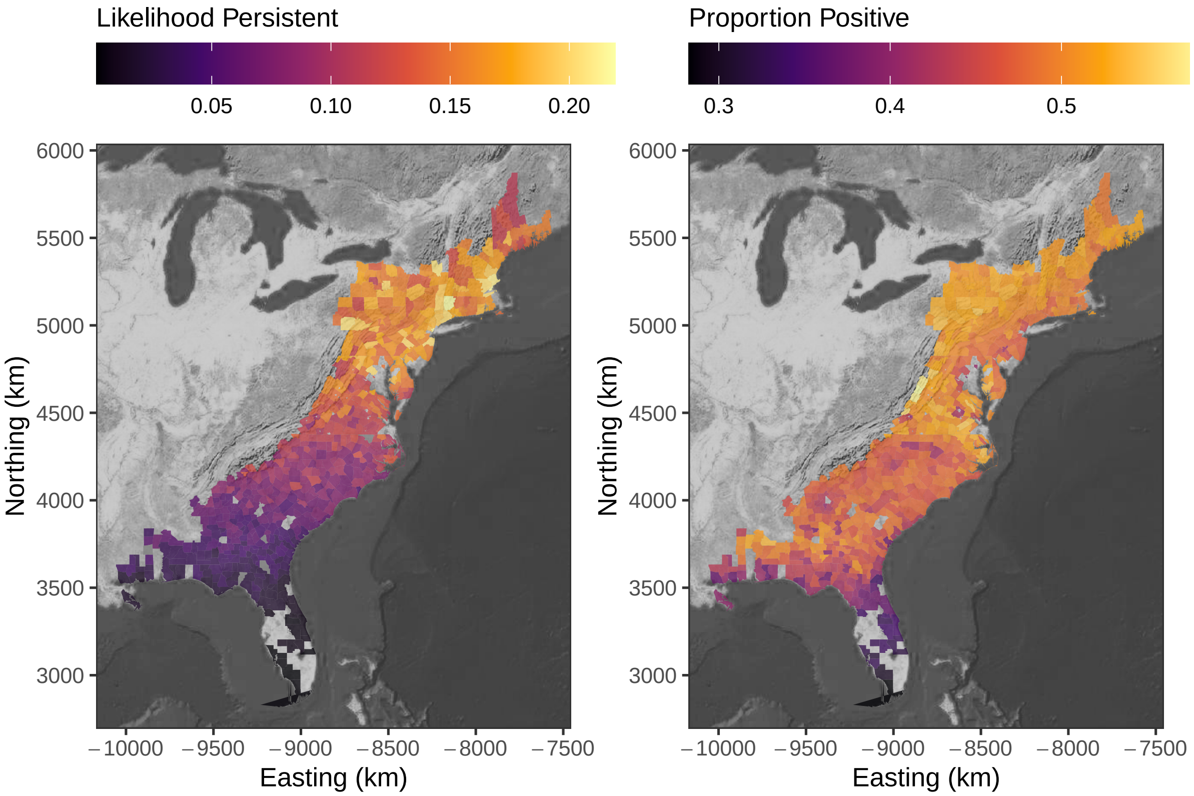

Figure D: Geographic distribution of model predictions (averaged across time) for persistence (likelihood of having at least 5 positive cases over the past 11 months; left), and proportion positive (proportion of all samples tested that are expected to be found positive; right).

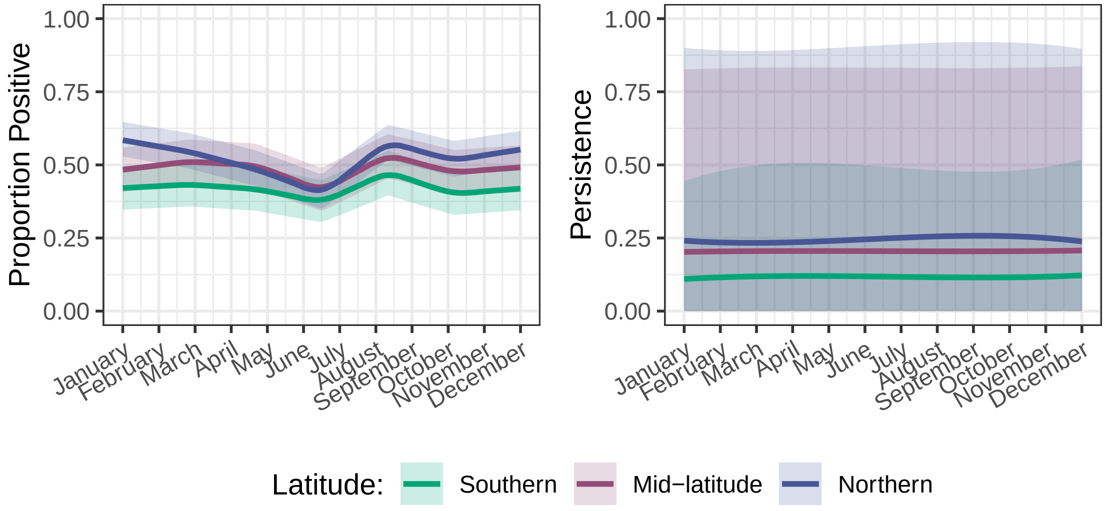

Figure E: Significant interaction between latitude and month on predicted rabies proportion positive and predicted likelihood of rabies persistence. The horizontal axis represents the month of the year, while the vertical axis is the model predicted proportion of samples submitted that will be found to be positive (top) or the predicted likelihood of rabies persistence (bottom). Three example latitudes spanning the south (30°N), mid-latitude (35°N), and north (40°N) are displayed. Confidence intervals depict the 10th to 90th quantile of model predictions for 1000 bootstrapped data points for each combination of latitude and month (see methods).

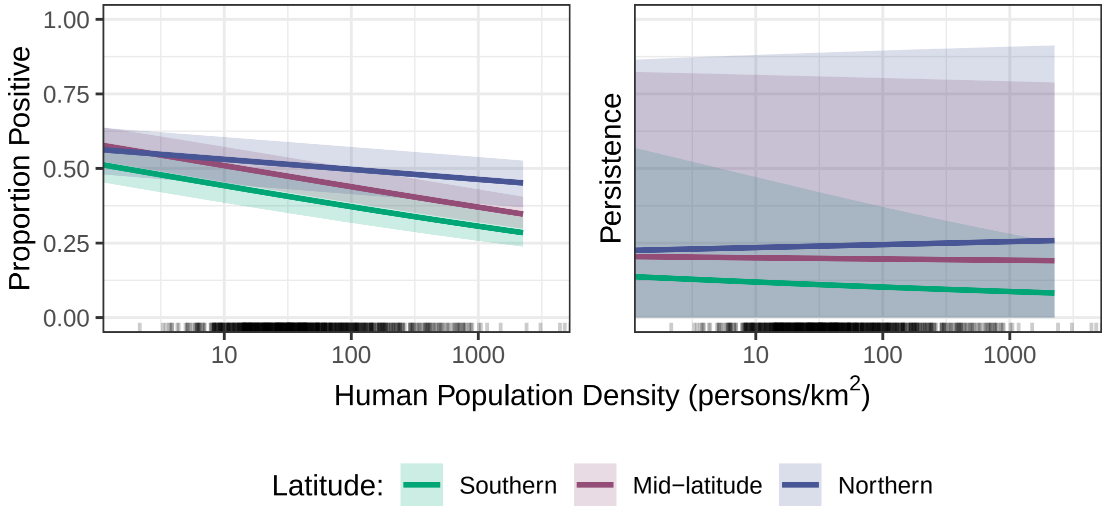

Figure F: Significant interaction between latitude and population density on predicted proportion of samples with positive results; non-significant relationship with predicted likelihood of rabies persistence. Human population density is plotted on the horizontal axis, including a rug to indicate where the counties used in this analysis fall in the range of densities. The vertical axis is the model predicted propotion of samples testing positive (top), and the predicted likelihood of a county having persistent rabies (bottom). Three example latitudes spanning the south (30°N), mid-latitude (35°N), and north (40°N) are displayed. Confidence intervals depict the 10th to 90th quantile of model predictions for 1000 bootstrapped data points for each combination of latitude and density (see methods).

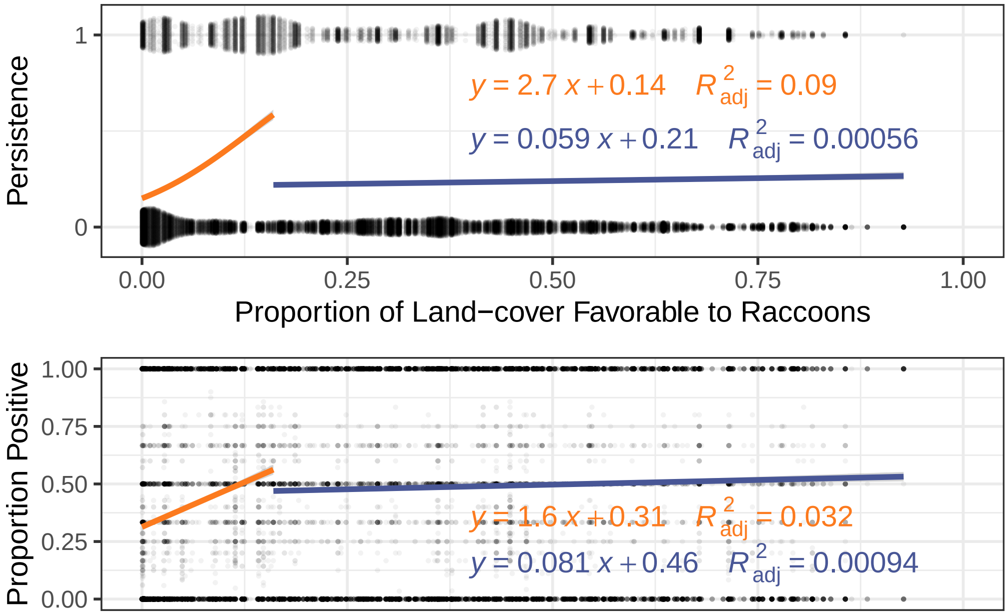

Figure G: Analogous to Figure 5 in the main text, favorable raccoon habitat is positively related to raccoon rabies proportion positive and persistence at very low levels of favorable habitat per county, but not at moderate-to-high levels. The raw data for the likelihood of a county having at least five months with at least one positive test each over the past 11 months ("persistence"; top) or the proportion of samples submitted that tested positive ("Proportion Positive"; bottom) is indicated with black points, which are jittered vertically for the binary measure of rabies persistence to indicate the frequency of counties with those habitat values. Two predicted lines are drawn for each response measure: an orange one which illustrates the positive relationship between habitat and outcome at low levels of raccoon-favorable habitat (less than 16% coverage), and a blue one illustrating that above a minimum threshold of available raccoon habitat, rabies is nearly independent of the exact area of favorable habitat. Note that, while the explained variance is low in all cases, the R^2 values at low raccoon-favorable habitat coverage is 161 and 34 times that at higher levels of raccoon-favorable habitat for persistence and proportion positive, respectively.

Summary of results for perisistence: Urbanness did not influence the likelihood of rabies persistence

Counties with 'persistent' raccoon rabies were defined as having at least 5 months with a rabid raccoon in the previous 11 months and approximately 25% of county-months in our dataset met this criteria. Raccoon rabies persistence was best explained by the significant effects of temporal lag, spatial effect, total number of samples over the lag period (which was 11 months), and an interaction term between latitude and month (seasonality that differed by latitude; Table D, Table B). Neither latitude nor population density were found to be significant predictors as main effects (Figure E, Figure F, Figure D). Because we averaged raccoon rabies cases over a broader time period, our outcome of persistence was a coarser measure than outcomes with the presence and proportion positive models.

Summary of results for proportion positive: Latitude, seasonality, and urbanness influence rabies proportion positive

We found that raccoon rabies proportion positive (the number of raccoons testing positive divided by the total number of raccoons submitted in that county-month) was likewise predicted by interactions of latitude with month and population density, as well as effects of temporal lag, spatial effect, and total number of samples submitted, differing only in the absence of a primary effect of population density (Table C, Table B).

The proportion of positive raccoons was higher in the north than in the south (Figure D, right), and in more rural areas compared to more urban areas (Figure E, Figure F, Figure D). More southern latitudes generally have less seasonal variation and lower proportion positive than northern latitudes (Figure E). Additionally, there is a negative relationship between proportion positive and population density, but with the effect of latitude becoming apparent only at more northern latitudes (at which point proportion positive was found to increase with latitude; Figure F).

Limitations when considering proportion positive on data from passive surveillance

County-months with no raccoons submitted were removed from our analysis, and those counties with low numbers of submitted raccoons could overinflate the importance of any rabies cases due to a simple combinatorial explanation (*i.e.*, with few tests, observed proportion positive options can take on only a few discrete values so when only one test is submitted in a given month, a positive test carries more weight than when there were many tests submitted) (Craft et al. 2009). This, along with potential biases in submission (*e.g.*, positive cases being more likely to be submitted than those without *a priori* suspicion, especially in places where rabies is not especially prevalent; (Pepin et al. 2017)) potentially could result in an over inflation of apparent raccoon rabies prevalence in the rural south. Finally, because both humans and raccoons are more active in the summer months, there is potentially a synergistic effect

on sample submissions, amplifying differences between observed prevalence patterns across seasons.

References

Craft, Meggan E, Erik Volz, Craig Packer, and Lauren Ancel Meyers. 2009. "Distinguishing Epidemic Waves from Disease Spillover in a Wildlife Population." *Proceedings of the Royal Society B: Biological Sciences* 276 (1663): 1777–85.

Pepin, Kim M, Amy J Davis, Daniel G Streicker, Justin W Fischer, Kurt C VerCauteren, and Amy T Gilbert. 2017. "Predicting Spatial Spread of Rabies in Skunk Populations Using Surveillance Data Reported by the Public." *PLoS Neglected Tropical Diseases* 11 (7): e0005822.
