# Supplementary material for: Landscape-scale analysis of raccoon rabies surveillance reveals different drivers of disease dynamics across latitude
Source: PLoS Negl Trop Dis. 2025 Sep 26;19(9):e0013581. doi: 10.1371/journal.pntd.0013581 (PMC12500145; doi:10.1371/journal.pntd.0013581)
Supplement: Attachment — Submitted filename: Response_to_Reviewers.pdf [file pntd.0013581.s004.pdf]

PNTD-D-24-01864: Landscape-scale analysis of raccoon rabies surveillance reveals different drivers of disease dynamics across latitude

Response to Reviewers

2025-08-05

Reviewer 1

We appreciate the reviewer's time and consideration of our work. We hope the reviewer aggrees that it is much improved as a result of the proposed revisions.

Methods

The paragraph (ll. 51-52) states that surveillance data from the 20 states is used for this analysis. It is recommended to clarify which states are being referred to. At the start of the summary, the authors refer to the Northeastern/mid-Atlantic US; however, I was unable to find a definition that matches the number of states. This may confuse other readers as well. Since it is important to understand the defined geographical area under surveillance, please consider describing this more clearly.

We now list all states from which data were collected at the beginning of the results section (l 261-264).

Regarding county-level data: what is the size of a county, and are the boundaries influenced by natural features, such as rivers? It would be interesting to understand if there was a difference in counties where borders were supported by natural structures regarding transmission. This raises questions about the impact of such features on inter-county species/viral spread.

We agree that terrain has a critical role to play in the spread of pathogens across the landscape. While some counties are delineated by natural terrain features, county shape and size is highly variable, both within and between states. Given the number of counties considered (634; 4134 pairwise borders), it is infeasible within

the scope of this study to evaluate which borders follow natural terrain features, anthropogenic features that likewise impede pathogen spread, or purely arbitrary political boundaries. Additionally some borders will be only partially described by impervious features, and not all such features are equally impermeable to pathogen spread. We now mention this important issue in our discussion of limitations (l 475-478).

Have the investigators considered assessing the geographical area under surveillance using a grid system along latitudes or categorizing the existing counties or other sector levels? This would allow them to weigh factors such as natural aspects (e.g., biodiversity, including areas rich in wildlife) and host diversity, including host species density (independent of urbanization density). It may also be useful to consider animal migration routes and climate (if applicable), comparing the different seasons that significantly differ across the states (e.g., is there an increase in cases in areas with longer winters versus areas with no or shorter winter periods?).

We appreciate the reviewer's close consideration of these key factors, which we agree are important to understanding disease spread through populations structured across complex landscapes. With respect to the particular suggestion of using a grid system, we agree this is a more systematic approach, however, given the limitations of our data (varying from specific locations to county-level location reporting), implementing a grid system, while making the analysis more standardized, would necessarily require making the spatial scale of the analysis more coarse than our current use of counties directly (the smallest possible conforming spatial scale). In our current analysis, we already consider a proxy for host density and climate, with migration being irrelevant in raccoons. Likewise, we consider differences in seasonality (defined as differences in outcome metrics across months) and possible interactions with latitudinal gradients.

It is mentioned (ll. 159-160) that "seasonality can increase spread through co-denning." I would suggest adding "viral" to clarify that seasonality increases the viral spread among raccoon populations due to their co-denning behavior.

Revised as suggested

Results

Surveillance Data: The surveillance dataset relies on enhanced sampling methods and passive surveillance, which means there is a high likelihood of underestimation. Even though the exclusion of counties with oral rabies vaccine (ORV) management is intended to minimize bias, it could also overlook or miss potentially indirect effects of these management practices on rabies dynamics in nearby areas (counties?) or in seasons without vaccination. A brief explanation of this exclusion reasoning would be beneficial to add, or acknowledging such considerations in the study limitations section.

We now mention this underestimation in the discussion (l 462-463), as well as clarify this omission in the methods (l 188-190)

Conclusions

The interactions between findings such as latitude/month and rabies presence and prevalence have been well discussed. Particularly, referring to “favorable habitat” in counties without raccoon density data was an interesting angle. Although the introduction indicates that the study results will provide further evidence to inform public health measures, very limited conclusions have been made so far. These valuable insights into disease dynamics could facilitate more informed suggestions for shaping education, surveillance, and management efforts.

We thank the reviewer for their close read, and agree our original submission was lacking in its connection between the promise of the abstract/introduction and the delivery in the discussion. We have added text throughout the discussion to better highlight how our results might be applied in the context of education, surveillance, and management.

Reviewer 2

Major comments

The study described in this manuscript addresses an important topic that will be of interest to readers of PNTD. The amount of data included in the study (~43,000 samples with ~18,000 rabid raccoons) is considerable, and the analysis methods applied are in general both appropriate and impressive.

The major weakness of the study is the data source: the data used is derived via passive surveillance, conducted at county- and state-level. Such surveillance data is likely subject to enormous bias in terms of the detection of cases and the sampling of detected cases, and as well as possibly the diagnosis of cases. The authors do acknowledge these biases in their discussion. However, the reader is left wondering if the results are valid, given the data source and it's known bias. I am not convinced that the results of this study provide insights into rabies occurrence, or if it is an analysis of rabies surveillance. As such, the finding that raccoon rabies in the eastern US is related to time, place and sampling effort confirms what is already known. Part of the problem is the inclusion of prevalence and persistence as outcomes of interest in this study. Both of these outcomes are very difficult to measure validly with the data available. Although I appreciate the authors intent for including these outcomes in addition to presence, they become a distraction from the analysis of presence data.

My suggestion is to simplify the analysis by restricting it to rabies presence, and then to more clearly make the argument why the type of data support the outcomes of that analysis.

We appreciate the reviewer's time and dedication to improving our work, and agree with many of the criticisms of our past presentation. We now simplify our presentation, focusing on the results for rabies presence and moving our other results to an extended supplement. As part of this simplification, we have also attempted to strengthen our arguments for the applicability of these data to understanding disease dynamics (beyond just understanding a surveillance process), with implications for education and management.

We hope the reviewer finds these revisions acceptable; we believe the work is much improved as a result of this process.

Specific comments

Abstract

Include some details of quantitative results in the abstract e.g. measures of autocorrelation, measures of association.

While we are limited to the extent we are able to detail our nuanced results in the abstract due to length constraints, we now include some more explicit descriptions of these results here and have thoroughly revised the main text.

Lines 62-64: what are these areas? Provide some more detailed information.

The specifics of these areas are provided in the previous sentences of the abstract, while this concluding sentence was intended as more of a general appeal for the value of long-term surveillance. We have added some additional details to the aforementioned sentences to more precisely detail our results.

Line 66: latitude is described as an environmental factor. Please check this – I consider it to be a geographical factor.

We have removed "environmental" to avoid confusion.

Lines 71-73: "best predicted by previous years' case data from the focal county or its neighboring counties". This finding is very focal, so what are the implications for rabies control? i.e. look where you've found it recently. This seems like an obvious and simplistic interpretation. More insights are needed to justify publication.

We have modified the language of this result; however, we also note that this excerpt is from the "Author Summary", which is written for non-experts, and is thus an intentionally simplified presentation. We provide more extensive, nuanced interpretations both in the abstract and in the main text of the work.

Lines 74-76: "Identification of trends in where and when cases are most likely to occur can help with a better understanding of the factors driving endemic disease dynamics in wildlife, and can help focus education, surveillance, and management efforts on higher risk areas and seasons." Based on this statement, knowing that raccoon rabies occurs where and when it has occurred before, and is dependent on the amount of sampling undertaken, the authors need to explain better how their current study meets this goal.

We have clarified our results in this author summary to better capture the details of our results, and have additionally reworked the main text discussion to highlight the implications of our results for targeting education, management, and surveillance.

Line 93: "the distribution of cases is far from uniform". This statement appears to be based on highly biased surveillance data; need to consider this before creating hypotheses for a phenomenon that might not exist.

We have modified the claim to be more precise, now noting that the "distribution of *confirmed* cases is far from uniform."

Lines 96-99: Unfortunately, it is very difficult (impossible?) to test hypotheses of disease dynamics with surveillance data, because surveillance would be expected to be driven largely by disease dynamics in the first place. Rabies is a classic example of this: when it is observed as

a clinical disease sampling occurs, when it is not there is little sampling done. This is the issue with using prevalence (the denominator problem) and persistence (haphazard surveillance) as outcomes.

We agree that surveillance effects are important to consider in disease modeling papers, especially in the case of surveillance that is responsive to disease prevalence, such that there are causal links between the two processes that make them difficult or impossible to disentangle. In some ways, this work is isolated from some of these concerns insofar as the sampling is passive and therfore relatively independent of observed disease prevalence. That is, there is not a systematic change in surveillance practice in response to a detected rabies case in a given county-month.

That is not to say that passive surveillance does not have its own complications (and we have re-worked our paper to better highlight these where appropriate). For instance, we agree the inconsistent sampling effort creates issues for our estimation of prevalence, and take the reviewer's advice to move this and persistence to an expanded Supporting Information We have also reworked the paper to hopefully make the distinction between surveillance and underlying disease dynamics more explicit (when possible) and to be more forthcoming about the limitations of the data sources for inference.

Introduction

The Introduction section is a bit long, and unfocused e.g. delving into vectors of disease (such as mosquito species), mange, rodent-borne pathogens. It is all interesting, but not necessarily relevant to the study conducted.

We have simplified and streamlined the introduction.

Methods

Lines 150-152: The passive surveillance data source is a major issue for results and conclusions

We agree this is a critical limitation to make explicit and have sought to do so throughout our revised draft.

Lines 154-157: "1) Presence: probability of at least one rabies case in raccoons (positive rabies sample), 2) Prevalence: the proportion of raccoon rabies surveillance samples that test positive, and 3) Persistence: the likelihood of persistent raccoon rabies occurrence (defined as at least n months with a positive sample over the last m months)." #2 and #3 are particularly biased measures. Considered whether it's best to focus on #1 to improve validity and interpretation.

As noted above, we take the reviewer's advice to focus on presence in the main text.

Lines 160-167: It is good to have the null hypotheses stated clearly! However, as per the above comments. These might need to be simplified.

The hypotheses have been simplified to fit with the more streamlined presentation.

Lines 173-175: Two different tests are included in the surveillance data that are used; is equivalent test accuracy assumed when tests are used in parallel? In other words, is there any differential diagnostic bias?

Both Direct Fluorescent Antibody (DFA) and Direct, Rapid Immunohistochemical (dRIT) Tests are approved and equivalent diagnostic methods (as described and supported by OIE/WOAH guidelines (Müller 2023) and the US-specific CSTE position statement ("Revision of Public Health Reporting and Timeframes for National Notification for Animal Rabies. Council of State and Territorial Epidemiologists" 2022)). We have now added this clarifying information to the Methods (l 160-161).

Line 178: county of collection is not equivalent to county of infection. Please justify that this is not an important source of bias.

While we agree the movement of animals and people across county lines makes this distinction necessary, we do not believe there is any reason to suspect the county of infection differs *systematically* from county of collection across our dataset, such that it would affect results at the scale of our analysis. Additionally, counties are quite large relative to average raccoon home range, reducing the likelihood of this type of mismatch occurring.

Line 179: in the case of two tests with discordant results, is the sample assumed to be positive? Please clarify.

We have elaborated on the testing protocols in the methods. In brief, samples are generally only submitted to repeated testing in instances of an inconclusive initial test result. As noted in the submitted methods, any samples that remained inconclusive in the dataset were dropped prior to analysis.

Lines 185-188: latitude and longitude of the sample location, in some states it was often the centroid of a county. There is an inconsistency in spatial accuracy of the data that requires clarification and justification.
Lines 197-199: Different county size and shapes; centroid does not represent the location adequately. Why not use actual lat/long when available to reduce this bias?

We agree that there are limitations to using county-level data, however, we believe using a nonhomegenous spatial resolution would introduce additional bias into our analysis. This is especially possible given that the spatial scale is different

across data sources, which is also the source of different tests. Thus, to increase interoperability between these two datasets as much as possible, latitudes were standardized to county centroids for all samples.

Lines 188-189: "we also removed any data with inconclusive rabies diagnostic test results." An analysis of the randomness of inconclusive test results would be helpful.

We now note that this only applies to 0.16% (68) of tests. Note that this only considers cases that were explicitly labeled as "inconclusive". Tests could additionally be considered inconclusive in other ways (e.g. in their typing), but we do not have a way to quantify this in our data. Such inconclusive typing was likewise omitted from the analysis.

Lines 190-195: "we focused our analysis on county-year combinations that had not been managed with the oral rabies vaccine (ORV) that year ... removed counties with rare detection, yet high-surveillance, at the edges of the current RABV range as detection probability was likely higher due to management and our intent was to focus on areas without management influence". These exclusions are problematic. It seems that the motivation is to only focus on "endemic" raccoon rabies areas, but using surveillance data is in itself, a circular argument. The results would have more credibility if some independent classification of endemic raccoon rabies areas was used, even if this means a substantial reduction in the amount of data analysed. As it is, seems like the data are manipulated to fit the analytical approach. There are also definitional questions – what is 'rare detection', what is 'high-surveillance' and what is 'current'? Again, it is worthwhile considering the focus of this study in terms of presence vs. persistence vs. incursion and spread. Disease management influence probably exists throughout the dataset. What are the effects of these data exclusions? Would need to see a sensitivity analysis to be convinced this approach is valid and reasonable.

We regret that we were not more explicit in detailing why these two counties were excluded from our analysis. In brief, they had exceptionally high levels of surveillance (more than 200 samples submitted in our dataset) while nonetheless not having even a single positive case detected. Moreover, these counties are at the western end of the raccoon rabies endemic range, and also near the southern extent of the ORV management zone. We now mention this in the methods, while also providing some additional context for the limitations of simply removing managed counties (l 182-188).

Regarding the use of the term "endemic," we agree there are limitations of language here. In the previous paragraph, we refer to the "endemic range," meaning the full spatial extent in which we see raccoon rabies cases. In contrast, in the previous submission (now in the Supporting Information), we are considering the "endemic" status of individual county-months, asking "is there rabies currently circulating (possibly undetected) in this county-month?" Historically, the National Rabies Surveillance System has classified counties as free from terrestrial

rabies if, "over the previous 5 years, they and any adjacent counties did not report any rabies cases and they tested 15 reservoir animals or 30 domestic animals" (Kunkel et al. 2023). However, this is clearly a very conservative measure, and simply taking its inverse is not appropriate to answer our opposite framing of the question (not "which counties are free from raccoon rabies", but "which counties have endemic raccoon rabies"). While far from perfect, our model-selection approach (detailed in the text and mentioned in a later response here) was intended to at least lend some justification to our chosen definition.

Line 199: The seasonality covariate was the month during which the sample was collected. This assumes a constant (and short) incubation period. Justify this assumption with some biological reasoning.

We believe this comment stems from a misunderstanding of our use of the term seasonality. In this work, we consider there to be a signature of seasonality when a response variable varies (predictably) throughout the year. For instance, if there are typically more cases detected in July than November, or vice versa. As such, we do not make any intrinsic assumptions about the incubation period of the pathogen except insofar as we assume it likewise does not vary seasonally.

We have attempted to clarify this interpretation in the methods. Moreover, we appreciate that our interpretation of the seasonality trends is a reflection of detection dates (rather than infection dates), and have attempted to make this nuance more clear as well (l 412-413).

Line 209: Given the county shape variation, total area of habitat should also have been evaluated in addition to proportion of the county containing raccoon favorable habitat.

When considering the absolute area of favorable habitat, rather than the proportion, we see a qualitatively similar result in R^2 (a 25-fold increase in explained variance for counties with less than 145 km^2 compared to those with greater than 145 km^2), but less distinction in slope.

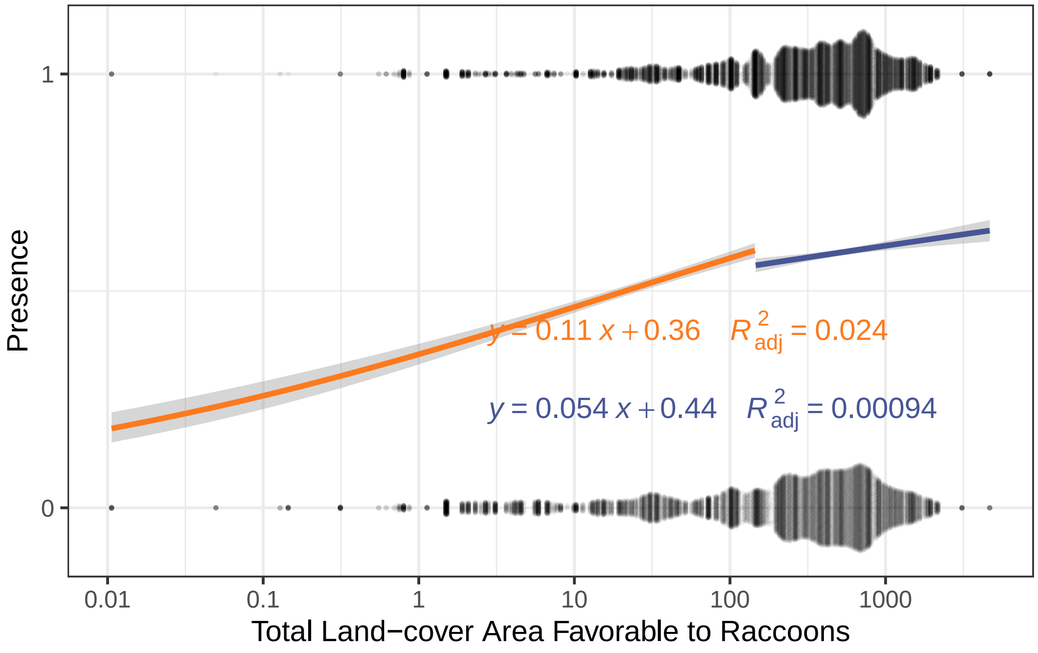

That said, we argue that, because we model raccoon rabies presence using a binomial model, where the success rate is a function of the trials (sample size), we are estimating a detection rate given effort. The proportion of the county containing raccoon favorable habitat therefore standardizes habitat similarly to our standardization of the response. Using total area, on the other hand results in a mismatch between the covariate and response. For this reason, we have opted to keep the figure axis as is for now.

Lines 215-216: Given the great variation in RABV infectious periods in raccoons (from 1 week to up to 19 weeks), perhaps some exploration of the temporal patterns in the data using time series analysis is warranted. Also, doesn't this mean there could be misclassification bias when using month of collection in the analysis?

We agree that a timeseries analysis would be a valuable contribution to the literature, but think this approach is too far outside of the scope of the present analysis. We have nonetheless added an additional figure to the Supporting Information (Figure C), showing that the slope of a temporal (i.e. using time as the independent variable) linear model fit to the total number of tests conducted, or positive test results, does not differ across latitudes. I.e. any change in testing intensity or disease prevalence throughout the thirteen years of our data does not differ systematically across latitudes.

Regarding potential misclassification bias due to variable incubation period, it is certainly possible that the month of detection does not match the month of infection, however, this difference is inconsistent, and unpredictable from the perspective of our dataset. We have added this potential source of bias to the

discussion, but we do not believe there is any reason to suspect this discrepancy to systematically bias our interpretations (l 478-482).

Lines 218-219: "(where high case reports in surrounding counties in the past might predict the number of current cases)" sounds more like surveillance bias than disease spread, but in reality, is impossible to disentangle. But this is a critical issue – are you analysing disease spread or surveillance effort?

We appreciate the reviewer highlighting this concern, which we agree is critical to the interpretation of our results. While we further agree that it is challenging to disentangle surveillance bias from disease spread with this dataset. Ultimately, however, we argue that our analysis has both implications for surveillance and insights for better understanding underlying disease dynamics. While sometimes frustrating, in practice, these two processes are necessarily treated as intertwined (e.g. Kunkel et al. (2023)).

Line 221: "(i.e., those sharing a border with the focal county)", which to be sounds like a geo-statistical rook contingency; a spatial distance buffer might be better, given the arrangement of county shapes.

We agree this is an important methodological choice in our work, with important implications for interpreting our results. While a spatial distance buffer makes the most sense for sufficiently spatially resolved data, we argue that applying this approach to county-aggregated data would introduce additional complications to our analysis while simultaneously being more computationally expensive to conduct.

For example, using a distance buffer would functionally make large counties more isolated from their neighbors than small counties. Likewise, layering a spatial buffer over spatially discrete data does not allow the model framework to match the ground truth of how these data are collected. Underlying both of these approaches is an understanding that diseases spread spatially, however because of the artificial county boundaries of data aggregation, a raccoon at one edge of one county is much more likely to transmit across that boundary than to counties that may have closer centroids on the other side. While our approach treats these as equally likely, at least it does not bias them in the opposite direction.

Model formulation

Line 228: "monthly apparent prevalence". How did you account for the (likely) great variation in the denominator?

We sought to deal with variation in the denominator both through the including the number of samples submitted as a fixed effect in the model construction and through considering a range of alternative outcome measures. Per the above

suggestions, we have moved these results to the Supporting Information and now focus on the detection of raccoon rabies presence, which is less sensitive to this concern.

Lines 233-238: definitions of persistence are confusing. My interpretation is that 2 out of 6 months would be defined as persistent, which seems a bit of a liberal definition. Is there any standard definition in this field that can be sued? If not, then some sort of sensitivity analysis should be undertaken, to observe if the definition makes a big difference to the analysis outcome. (Only of course if you plan to retain the persistence analysis in this manuscript).

There has been some mis-communication here. Our definition for persistence was determined to be at least 5 months with positive test results out of the past 11 months. As noted earlier, unfortunately, there is not a standard definition for this value in the field. Due to the limitations of passive surveillance and the complications of a disease with a highly variable, long incubation period, some more intuitive results (like consecutive months with positive tests) are not viable.

As a result, we used a model selection approach (previously detailed on lines 233-238, with results on lines 375-377, now in the Supporting Information), in which we performed a constrained search to find the optimal number of months for each of the number positive and the window over which to search, selecting the most explanatory combination.

We have moved these methods and results to the Supporting Information, and we hope the explanation here, as well as the relocation, clears up some of the confusion on this point.

Lines 238-241: "We use the statistically appropriate term 'apparent' throughout this paragraph to highlight that these response variables are biased samples of the 'true' presence, prevalence, or persistence because our analysis does not account for error in the observation process." This is a big problem! Really, you should only be using the term "proportion positive". With the surveillance data you have you cannot estimate prevalence, so that use of this term is misleading.

We have adjusted the language for prevalence to read "proportion positive" and have moved these results to the Supporting Information as requested.

Line 243: including the total number of tests per county per month partly controls for sampling, but at a monthly level testing will fluctuate a lot. But this also makes the findings with respect to the log(Total Number of Tests Conducted + 1) variable difficult to interpret. Or does this sentence mean that sampling was included (forced into) all models as an attempt to adjust for sampling? This needs to be clarified.

We included the (log) total number of monthly samples submitted (i.e. sampling intensity) into all models in the form of a fixed effect in the GLMM. Thus, each

county-month's prediction incorporates the sampling intensity taking place in that county in that month.

We have added a clause to make this more explicit in the methods (l 231-232)

Results

Novelty of results. I feel that this tells us only about the surveillance effort i.e. modelling rabies surveillance, not rabies occurrence. The two are not the same, due to sampling bias.

We agree that it is important to distinguish between the effects of surveillance and those of the underlying disease dynamics. While it is true that the passive nature of our data limits some of our inference, we do not agree that we are precluded from considering the disease implications in conjunction with those for surveillance. Moreover, understanding patterns that emerge from surveillance can be useful for evaluating the efficiency of ongoing surveillance and adapting it for different management objectives.

We have attempted to improve the legibility of our discussion of this nuance in the work, better highlighting limitations, while also discussing places where we have taken steps to mitigate the consequences of the intrinsic biases of passive surveillance.

We hope that the revised manuscript mitigates some of the reviewer's concerns, while also providing some insight into raccoon rabies dynamics in the eastern United States.

Lines 292-293: "Thus, in the north more samples are submitted in the summer months when raccoon rabies is less likely to be detected." This is a peculiar finding that is specific to prevalence, and I suspect it is an artifact of the passive surveillance data that us used in this study.

We agree this result is heavily influenced by the passive nature of our surveillance data. We have attempted to make this connection more explicit in our discussion of the limitations of using passive surveillance to estimate prevalence in our Supporting Information.

Lines 351-353: "At high population densities ... we see southern and northern counties converging to have higher raccoon rabies detection probabilities than more central counties." This is an important observation for control and surveillance, and perhaps this study is more suited to making recommendations for surveillance than for understanding rabies epidemiology in this region.
Lines 368-369: "we found a general negative relationship between prevalence and population density". Again, there are implications for rabies surveillance, rather than insights into rabies epidemiology.

Lines 431-435: "However, in the north, more raccoons were submitted for rabies surveillance in the summer compared to the winter; therefore, inferring case trends from looking only at the number of positive samples through time could be misleading. In the north, more raccoons might be submitted in the summer than in the winter because humans are more likely to be outside in the summer." Again, surveillance implications versus rabies epidemiology insights. Lines 445-447: "The surprise here was the notable drop in raccoon rabies presence in the summer in the far north, despite increased sampling". I agree, surprising! Implications for surveillance.

We group these four comments together for their thematic similarity. In response to this concern, we have added more surveillance implications to the discussion. For example, we have now focused on improving efficiency of surveillance through prioritizing testing of higher risk cases (l 382-389), improving the targetting of educational resources (l 427-429), and improving our discussion of the limitations of using passive sampling to understand disease dynamics (l 458-467).

Lines 374-376: "Counties with 'persistent' raccoon rabies were defined as having at least 5 months with a rabid raccoon in the previous 11 months and approximately 25% of county-months in our dataset met this criterion." If persistence remains as an outcome in this study, then it is important to clarify and justify the definition that is used. This is especially important given the acknowledgement that 'Because we averaged raccoon rabies cases over a broader time period, our outcome of persistence was a coarser measure than outcomes with the presence and prevalence models.'

We have moved these results and their associated methods to the Supporting Information.

Lines 383-393: "Although the relationships between raccoon favorable habitat and presence, prevalence, and persistence were not statistically significant in the top models, we did find an interesting threshold effect." I don't see value in drawing these conclusions given that the analysis was not statistically significant. This section should be deleted.

While we did not see a significant effect across the whole range of habitat values, we still believe the piecewise analysis has value, and piecewise relationships are not able to be captured by standard GLMM approaches. We now make this argument more explicitly, as well as highlight why we think this result is worth including in the paper in the results and discussion.

Discussion

Lines 407-410: "We find that county-level raccoon rabies dynamics are strongly driven by temporal ... and spatial ... context, as well as testing intensity ... . As above, the importance of these results is more to do with surveillance than rabies epidemiology.

While we agree that testing intensity in particular is independent of disease processes (and therefore more of an indication of surveillance dynamics), temporal and spatial patterns of disease detection are a product of both surveillance and underlying disease dynamics. Moreover, these methods are in line with current CDC policies which use apparent prevalence values to guide recommendations (Kunkel et al. 2023). We hope our re-worked manuscript makes this distinction more clear.

Lines 410-413: latitude and urbanness seem to play a relatively minor role once time, space and sampling taken into account. This conclusion does not seem to come through clearly in the manuscript.

We regret any implication we have made regarding relative feature importance, as this is non-trivial to calculate in the case of GLMMs, especially when fitting spline parameters and interactions between those splines, as we do here. To reduce confusion on this point, we have changed "best predicted" to "heavily influenced" in our conclusion. We have additionally re-worked our presentation to hopefully not oversell our results.

A weakness of the analysis is the lack of a population at-risk. I'd like to see a sensitivity analysis of this, do results changed if population distribution maps change?

We are not sure what the reviewer means by this comment. We include human population density as a fixed effect in the model, and represent the detected relationship in Fig 4. Sensitivity to empirical relationships between fixed variables is captured through the confidence intervals in our figures, which are produced by permuting the data such that fixed effects are effectively decoupled (see methods for details).

Lines 452-456: "In our analysis, we aggregated across years, possibly obscuring an effect of the time-since-introduction, which varies across the spatial extent of our data. In particular, one might expect that the relatively recent introduction of rabies to the northern states might be consequential to disease dynamics. We do not see any evidence for this in our data." But weren't the areas where rabies had been recently introduced excluded from data analysis? So, I find this statement to be confusing.

We apologize for the confusion on this point, by "relatively recent introduction," we were referring to the difference between the mid-atlantic/northeastern states (where rabies was introduced in the 1970's) and those in the south, where rabies was endemic before then. We have clarified this sentence.

Lines 469-470: "What was contrary to our expectations was the highest raccoon rabies prevalence rates in the counties with the lowest human population density (rural areas), especially in the south." This could well be the "denominator effect". The analysis of prevalence with the data available (even the use of the term) is very problematic.

In addition to moving these results to the Supporting Information (as noted in previous responses), we have also moved all relevant discussion of limitations, including those related to the denominator (previously lines 473-479).

Lines 482-483: Our counterintuitive urbanness results might have to do with challenging sampling issues ... counties with more people had more raccoons submitted for testing. Many of findings might be explained by sampling bias.

We agree that sampling biases are an important limitation to our work. We attempt to alleviate some of these concerns by including the (log) number of samples submitted in our model construction to account for variation in sampling intensity, but have also better highlighted these concerns in our revised draft (l 457-469).

Lines 496-498: "i.e., with few tests, observed prevalence options can take on only a few discrete values so when only one test is submitted in a given month, a positive test carries more weight than when there were many tests submitted." Exactly! Hence my suggestion not to include prevalence in your analysis.

As recommended, we have moved all results other than presence to the Supporting Information.

What about the lack of independence? i.e. once one rabid case identified there is more sampling?

We regret this point was not made more clear in our original submission. While it is possible there is some limited responsiveness to detecting a positive raccoon (e.g. through local communication within a county), the bulk of this sampling, and especially sampling up to the first positive case, is passive. That is, though infected animals are perhaps more likely to be found dead and/or reported, there is very limited responsiveness to previous test results in the form of increased sample submission. We now state this explicitly (l 478-479).

Lines 517-519: It is still not entirely clear to me whether the null hypotheses have been rejected or not. "ultimately we did find a trend with latitude" (but not urbanness?) feels to me like more is being read into the results than is appropriate. If we only focus on presence, then rabies presence is associated with time, place and sampling.

We apologize that this sentence was not as clear as it could have been, though we believe the final sentence might stem from a misreading of Table 1. We have reworked this sentence to A) focus on the results of rabies presence, B) more clearly distinguish that we found a significant interaction between urbanness and latitude, and C) note that this interaction was both less expected and more complex than the temporal, spatial, and sampling effects. We hope the revised sentence is improved in both comprehension and more true to our interpretation of the results.

Figures

Figure 2: Grey shading is difficult to visualise. Essentially there seems to be no variation in the estimates, presumably because of the large sample size.

We have removed the grey shading to simplify this figure since variation is already shown with the overlaid data points.

Figure 3: 'presence' the only outcome with apparent seasonality, but it is also highly variable.

Our results suggest a role of seasonality (defined as a significant effect of month, possibly through an interaction term) for all three outcome variables, with visible month-to-month deviations in both presence and percent positive in the submitted Figure 2. The absence of a visible effect for persistence is expected based on the definition spanning 11 months, as noted in our original submission.

Nonetheless, we believe this issue is mitigated by the proposed (and executed) re-arrangement, moving all results other than those related to presence to the Supporting Information.

Figure 5: Extremely small R2 values, what does this mean? Is it useful to show?

We agree that habitat is not a major predictor of rabies presence. As noted in previous responses, we nevertheless still believe the piecewise analysis has value, especially as a means to stimulate thought about more complex relationships that might not be captured in this modelling framework. Regarding the R^2 values in particular, our focus is more on the contrast in R^2 between the two regions of parameter space (rather than the absolute values *per se*. We now make this explicit in the caption (l 371-375).

References

Kunkel, Amber, Gabriella Veytsel, Sarah Bonaparte, Haillie Meek, Xiaoyue Ma, Amy J. Davis, Jesse Bonwitt, and Ryan M. Wallace. 2023. "Defining County-Level Terrestrial Rabies Freedom Using the US National Rabies Surveillance System: Surveillance Data Analysis." *JMIR Public Health and Surveillance* 9 (1): e43061. https://doi.org/10.2196/43061.

Müller, Thomas. 2023. "Rabies (Infection with Rabies Virus and Other Lyssaviruses): Chapter 3.1.19." In *Manual of Diagnostic Tests and Vaccines for Terrestrial Animals 2023: WOAH Terrestrial Manual 2023*.

"Revision of Public Health Reporting and Timeframes for National Notification for Animal Rabies. Council of State and Territorial Epidemiologists." 2022. https://cdn.ymaws.com/www.cste.org/resource/resmgr/ps/ps2022/22-ID-06_Animal_Rabies.pdf.
