# Supplementary material for: Landscape-scale analysis of raccoon rabies surveillance reveals different drivers of disease dynamics across latitude
Source: PLoS Negl Trop Dis. 2025 Sep 26;19(9):e0013581. doi: 10.1371/journal.pntd.0013581 (PMC12500145; doi:10.1371/journal.pntd.0013581)
Supplement: Attachment — Submitted filename: PNTD-D-24-01864_review.docx [file pntd.0013581.s002.docx]

| **Manuscript Number:** | PNTD-D-24-01864 |
| --- | --- |
| **Full Title:** | Landscape-scale analysis of raccoon rabies surveillance reveals different drivers of disease dynamics across latitude |
| **Short Title:** | Raccoon rabies dynamics across latitude and urbanness |
| **Article Type:** | Research Article |

Major comments

The study described in this manuscript addresses an important topic that will be of interest to readers of PNTD. The amount of data included in the study (~43,000 samples with ~18,000 rabid raccoons) is considerable, and the analysis methods applied are in general both appropriate and impressive.

The major weakness of the study is the data source: the data used is derived via passive surveillance, conducted at county- and state-level. Such surveillance data is likely subject to enormous bias in terms of the detection of cases and the sampling of detected cases, and as well as possibly the diagnosis of cases. The authors do acknowledge these biases in their discussion. However, the reader is left wondering if the results are valid, given the data source and it’s known bias. I am not convinced that the results of this study provide insights into rabies occurrence, or if it is an analysis of rabies surveillance. As such, the finding that raccoon rabies in the eastern US is related to time, place and sampling effort confirms what is already known. Part of the problem is the inclusion of prevalence and persistence as outcomes of interest in this study. Both of these outcomes are very difficult to measure validly with the data available. Although I appreciate the authors intent for including these outcomes in addition to presence, they become a distraction from the analysis of presence data.

My suggestion is to simplify the analysis by restricting it to rabies presence, and then to more clearly make the argument why the type of data support the outcomes of that analysis.

Specific comments

**Abstract**

Include some details of quantitative results in the abstract e.g. measures of autocorrelation, measures of association.

Lines 62-64: what are these areas? Provide some more detailed information.

Line 66: latitude is described as an environmental factor. Please check this – I consider it to be a geographical factor.

Lines 71-73: “best predicted by previous years’ case data from the focal county or its neighboring counties”. This finding is very focal, so what are the implications for rabies control? i.e. look where you've found it recently. This seems like an obvious and simplistic interpretation. More insights are needed to justify publication.

Lines 74-76: “Identification of trends in where and when cases are most likely to occur can help with a better understanding of the factors driving endemic disease dynamics in wildlife, and can help focus education, surveillance, and management efforts on higher risk areas and seasons.“ Based on this statement, knowing that raccoon rabies occurs where and when it has occurred before, and is dependent on the amount of sampling undertaken, the authors need to explain better how their current study meets this goal.

Line 93: “the distribution of cases is far from uniform”. This statement appears to be based on highly biased surveillance data; need to consider this before creating hypotheses for a phenomenon that might not exist.

Lines 96-99: Unfortunately, it is very difficult (impossible?) to test hypotheses of disease dynamics with surveillance data, because surveillance would be expected to be driven largely by disease dynamics in the first place. Rabies is a classic example of this: when it is observed as a clinical disease sampling occurs, when it is not there is little sampling done. This is the issue with using prevalence (the denominator problem) and persistence (haphazard surveillance) as outcomes.

The Introduction section is a bit long, and unfocused e.g. delving into vectors of disease (such as mosquito species), mange, rodent-borne pathogens. It is all interesting, but not necessarily relevant to the study conducted.

**Methods**

Lines 150-152: The passive surveillance data source is a major issue for results and conclusions

Lines 154-157: “1) Presence: probability of at least one rabies case in raccoons (positive rabies sample), 2) Prevalence: the proportion of raccoon rabies surveillance samples that test positive, and 3) Persistence: the likelihood of persistent raccoon rabies occurrence (defined as at least *n* months with a positive sample over the last *m* months).” #2 and #3 are particularly biased measures. Considered whether it’s best to focus on #1 to improve validity and interpretation.

Lines 160-167: It is good to have the null hypotheses stated clearly! However, as per the above comments. These might need to be simplified.

Lines 173-175: Two different tests are included in the surveillance data that are used; is equivalent test accuracy assumed when tests are used in parallel? In other words, is there any differential diagnostic bias?

Line 178: county of collection is not equivalent to county of infection. Please justify that this is not an important source of bias.

Line 179: in the case of two tests with discordant results, is the sample assumed to be positive? Please clarify.

Lines 185-188: latitude and longitude of the sample location, in some states it was often the centroid of a county. There is an inconsistency in spatial accuracy of the data that requires clarification and justification.

Lines 188-189: “we also removed any data with inconclusive rabies diagnostic test results.” An analysis of the randomness of inconclusive test results would be helpful.

Lines 190-195: “we focused our analysis on county-year combinations that had not been managed with the oral rabies vaccine (ORV) that year … removed counties with rare detection, yet high-surveillance, at the edges of the current RABV range as detection probability was likely higher due to management and our intent was to focus on areas without management influence”. These exclusions are problematic. It seems that the motivation is to only focus on “endemic” raccoon rabies areas, but using surveillance data is in itself, a circular argument. The results would have more credibility if some independent classification of endemic raccoon rabes areas was used, even if this means a substantial reduction in the amount of data analysed. As it is, seems like the data are manipulated to fit the analytical approach. There are also definitional questions – what is ‘rare detection’, what is ‘high-surveillance’ and what is ‘current’? Again, it is worthwhile considering the focus of this study in terms of presence vs. persistence vs. incursion and spread. Disease management influence probably exists throughout the dataset. What are the effects of these data exclusions? Would need to see a sensitivity analysis to be convinced this approach is valid and reasonable.

Lines 197-199: Different county size and shapes; centroid does not represent the location adequately. Why not use actual lat/long when available to reduce this bias?

Line 199: The seasonality covariate was the month during which the sample was collected. This assumes a constant (and short) incubation period. Justify this assumption with some biological reasoning.

Line 209: Given the county shape variation, total area of habitat should also have been evaluated in addition to proportion of the county containing raccoon favorable habitat.

Lines 215-216: Given the great variation in RABV infectious periods in raccoons (from 1 week to up to 19 weeks), perhaps some exploration of the temporal patterns in the data using time series analysis is warranted. Also, doesn’t this mean there could be misclassification bias when using month of collection in the analysis?

Lines 218-219: “(where high case reports in surrounding counties in the past might predict the number of current cases)” sounds more like surveillance bias than disease spread, but in reality, is impossible to disentangle. But this is a critical issue – are you analysing disease spread or surveillance effort?

Line 221: “(i.e., those sharing a border with the focal county)”, which to be sounds like a geostatistical rook contingency; a spatial distance buffer might be better, given the arrangement of county shapes.

**Model formulation**

Line 228: “monthly apparent prevalence”. How did you account for the (likely) great variation in the denominator?

Lines 233-238: definitions of persistence are confusing. My interpretation is that 2 out of 6 months would be defined as persistent, which seems a bit of a liberal definition. Is there any standard definition in this field that can be sued? If not, then some sort of sensitivity analysis should be undertaken, to observe if the definition makes a big difference to the analysis outcome. (Only of course if you plan to retain the persistence analysis in this manuscript).

Lines 238-241: “We use the statistically appropriate term ‘apparent’ throughout this paragraph to highlight that these response variables are biased samples of the ‘true’ presence, prevalence, or persistence because our analysis does not account for error in the observation process.” This is a big problem! Relly, you should only be using the term “proportion positive”. With the surveillance data you have you cannot estimate prevalence, so that use of this term is misleading.

Line 243: including the total number of tests per county per month partly controls for sampling, but at a monthly level testing will fluctuate a lot. But this also makes the findings with respect to the log(Total Number of Tests Conducted + 1) variable difficult to interpret. Or does this sentence mean that sampling was included (forced into) all models as an attempt to adjust for sampling? This needs to be clarified.

**Results**

Novelty of results. I feel that this tells us only about the surveillance effort i.e. modelling rabies surveillance, not rabies occurrence. The two are not the same, due to sampling bias.

Lines 292-293: “Thus, in the north more samples are submitted in the summer months when raccoon rabies is less likely to be detected.” This is a peculiar finding that is specific to prevalence, and I suspect it is an artifact of the passive surveillance data that us used in this study.

Lines 351-353: “At high population densities … we see southern and northern counties converging to have higher raccoon rabies detection probabilities than more central counties.” This is an important observation for control and surveillance, and perhaps this study is more suited to making recommendations for surveillance than for understanding rabies epidemiology in this region.

Lines 368-369: “we found a general negative relationship between prevalence and population density”. Again, there are implications for rabies surveillance, rather than insights into rabies epidemiology.

Lines 374-376: “Counties with ‘persistent’ raccoon rabies were defined as having at least 5 months with a rabid raccoon in the previous 11 months and approximately 25% of county-months in our dataset met this criterion.” If persistence remains as an outcome in this study, then it is important to clarify and justify the definition that is used. This is especially important given the acknowledgement that ‘Because we averaged raccoon rabies cases over a broader time period, our outcome of persistence was a coarser measure than outcomes with the presence and prevalence models.’

Lines 383-393: “Although the relationships between raccoon favorable habitat and presence, prevalence, and persistence were not statistically significant in the top models, we did find an interesting threshold effect.” I don’t see value in drawing these conclusions given that the analysis was not statistically significant. This section should be deleted.

**Discussion**

Lines 407-410: “We find that county-level raccoon rabies dynamics are strongly driven by temporal … and spatial … context, as well as testing intensity … . As above, the importance of these results is more to do with surveillance than rabies epidemiology.

Lines 410-413: latitude and urbanness seem to play a relatively minor role once time, space and sampling taken into account. This conclusion does not seem to come through clearly in the manuscript.

A weakness of the analysis is the lack of a population at-risk. I’d like to see a sensitivity analysis of this, do results changed if population distribution maps change?

Lines 431-435: “However, in the north, more raccoons were submitted for rabies surveillance in the summer compared to the winter; therefore, inferring case trends from looking only at the number of positive samples through time could be misleading. In the north, more raccoons might be submitted in the summer than in the winter because humans are more likely to be outside in the summer.” Again, surveillance implications versus rabes epidemiology insights.

A weakness of the analysis - population at-risk. Would like to see a sensitivity analysis of this, do results changed if distribution maps change?

Lines 445-447: “The surprise here was the notable drop in raccoon rabies presence in the summer in the far north, despite increased sampling”. I agree, surprising! Implications for surveillance.

Lines 452-456: “In our analysis, we aggregated across years, possibly obscuring an effect of the time-since-introduction, which varies across the spatial extent of our data. In particular, one might expect that the relatively recent introduction of rabies to the northern states might be consequential to disease dynamics. We do not see any evidence for this in our data.” But weren’t the areas where rabies had been recently introduced excluded from data analysis? So, I find this statement to be confusing.

Lines 469-470: “What was contrary to our expectations was the highest raccoon rabies prevalence rates in the counties with the lowest human population density (rural areas), especially in the south.” This could well be the “denominator effect”. The analysis of prevalence with the data available (even the use of the term) is very problematic.

Lines 482-483:

Our counterintuitive urbanness results might have to do with challenging sampling issues … counties with more people had more raccoons submitted for testing. Many of findings might be explained by sampling bias.

Lines 496-498: “i.e., with few tests, observed prevalence options can take on only a few discrete values so when only one test is submitted in a given month, a positive test carries more weight than when there were many tests submitted.” Exactly! Hence my suggestion not to include prevalence in your analysis.

What about the lack of independence? i.e. once one rabid case identified there is more sampling?

Lines 517-519: It is still not entirely clear to me whether the null hypotheses have been rejected or not. “ultimately we did find a trend with latitude” (but not urbanness?) feels to me like more is being read into the results than is appropriate. If we only focus on presence, then rabies presence is associated with time, place and sampling.

**Figure 2**

Grey shading is difficult to visualise. Essentially there seems to be no variation in the estimates, presumably because of the large sample size.

**Figure 3**

‘presence’ the only outcome with apparent seasonality, but it is also highly variable.

**Figure 5**

Extremely small R2 values, what does this mean? Is it useful to show?
